# Zuo-Gui-Wan Aqueous Extract Ameliorates Glucocorticoid-Induced Spinal Osteoporosis of Rats by Regulating let-7f and Autophagy

**DOI:** 10.3389/fendo.2022.878963

**Published:** 2022-05-03

**Authors:** Gengyang Shen, Qi Shang, Zhida Zhang, Wenhua Zhao, Honglin Chen, Ibrayinjan Mijiti, Guifeng Chen, Xiang Yu, Fuyong Yu, Peng Zhang, Jiahui He, Xuelai Zhang, Jingjing Tang, Jianchao Cui, De Liang, Lingfeng Zeng, Hui Ren, Xiaobing Jiang

**Affiliations:** ^1^Department of Spinal Surgery, The First Affiliated Hospital of Guangzhou University of Chinese Medicine, Guangzhou, China; ^2^Lingnan Medical Research Center of Guangzhou University of Chinese Medicine, Guangzhou, China; ^3^Department of Spinal Surgery, Nanshan Hospital, The First Affiliated Hospital of Guangzhou University of Chinese Medicine, Shenzhen Nanshan Hospital of Chinese Medicine, Guangzhou, China; ^4^The First Clinical College, Guangzhou University of Chinese Medicine, Guangzhou, China; ^5^Department of Spinal Surgery, The First People’s Hospital of Kashgar, Kashgar, China; ^6^Department of Orthopedics, The 2nd Affiliated Hospital of Guangzhou University of Chinese Medicine, Guangzhou, China

**Keywords:** ZGW aqueous extract, GIOP, let-7f, autophagy, Chinese medicine

## Abstract

**Objective:**

This study proposes to explore the protective effect of Zuo-Gui-Wan (ZGW) aqueous extract on spinal glucocorticoid-induced osteoporosis (GIOP) *in vivo* and *in vitro*, and the underlying mechanisms of ZGW in GIOP and osteogenic differentiation of bone marrow-derived mesenchymal stem cells (BMSCs) were conducted.

**Methods:**

*In vivo*, SD rats were randomly divided into three groups: control group (CON), dexamethasone (DEXM) group, and ZGW group, which were given vehicle, DEXM injection, and ZGW intragastric administration at the same time. Vertebral bone microarchitecture, biomechanics, histomorphology, serum AKP activity, and the autophagosome of osteoblasts were examined. The mRNA expressions of let-7f, autophagy-associated genes (mTORC1, Beclin-1, ATG12, ATG5, and LC3), Runx2, and CTSK were examined. *In vitro*, the let-7f overexpression/silencing vector was constructed and transfected to evaluate the osteogenic differentiation of BMSCs. Western blot was employed to detect the expression of autophagy-associated proteins (ULK2, ATG5, ATG12, Beclin-1, LC3).

**Results:**

*In vivo*, ZGW promoted the bone quantity, quality, and strength; alleviated histological damage; increased the serum AKP activity; and reduced the autophagosome number in osteoblasts. Moreover, ZGW increased the let-7f, mTORC1, and Runx2 mRNA expressions and reduced the Beclin-1, ATG12, ATG5, LC3, and CTSK mRNA expressions. *In vitro*, bioinformatics prediction and dual luciferase reporter gene assay verified that let-7f targeted the binding to ULK2 and negatively regulated the ULK2 expression. Furthermore, by let-7f overexpression/silencing, ZGW may promote osteoblast differentiation of BMSCs by regulating let-7f and autophagy as evidenced by Western blot (ULK2, ATG5, ATG12, Beclin-1, LC3).

**Conclusions:**

ZGW may ameliorate GC-induced spinal osteoporosis by promoting osteoblast differentiation of BMSCs by activation of let-7f and suppression of autophagy.

## Introduction

Glucocorticoid-induced osteoporosis (GIOP) is the most common secondary osteoporosis, which is characterized by decreased bone mass and deteriorated bone microarchitecture and affects as many as 50% of patients who receive therapy with chronic glucocorticoid (GCs), the commonly used anti-inflammatory drugs (1). More and more pharmacological treatments, including calcium, vitamin D, bisphosphonates, teriparatide, and denosumab, have been recommended for treating osteoporosis, whereas GIOP is still clinically not well treated due to the side effects and “drug holidays” of existing management (1, 2). Therefore, a new drug therapy against GIOP is still supposed to be developed.

Traditional Chinese medicine (TCM) has fought against bone metabolism-associated diseases for more than 1,000 years and has accumulated a wealth of herbal compounds and clinical practice experience (3–5). As a well-known TCM for treating GIOP, Zuo-Gui-Wan (ZGW) comprises eight herbal medicines: Radix Rehmanniae, Semen Cuscutae, Radix Achyranthis Bidentatae, Carapax et Plastrum Testudinis, Cornu Cervi Pantotrichum, Rhizoma Dioscoreae, Fructus Corni, and Fructus Lycii. Recent research has found that ZGW can treat ovariectomy-induced osteopenia by regulating the Th17/Treg or RANKL/OPG signal transduction pathway (6–9). Moreover, our previous studies have demonstrated that extracts from plastrum testudinis, one of the major active ingredients of ZGW, may ameliorate GIOP *via* regulating OPG, Runx2, and CTSK expressions (10, 11). However, the pharmacological effect and mechanism of ZGW treating GIOP are still uncomprehensive and unclear.

MicroRNAs (miRNAs) consist of 19–25 nucleotides and regulate gene expressions at the posttranscriptional level by binding to 3′UTRs of target mRNAs (12). Accumulating evidence points to an intimate connection between miRNAs and bone metabolism, keeping balanced by osteoblastic bone formation and osteoclastic bone resorption (12, 13). However, the studies about the action mechanisms of miRNAs on GIOP are still limited. Our previous study has demonstrated that miRNA let-7f mimics could ameliorate GIOP (14), but the underlying mechanisms are still not elucidated. Therefore, the alterations in miRNA expression levels are associated with the pathogenesis of GIOP, but the underlying signaling mechanisms are still not well characterized and further studies are required.

Autophagy is characterized by the formation of autophagosomes by cell membrane encapsulation of part of the cytoplasm and organelles and fusion with lysosomes to form autophagolysosomes to degrade the encapsulated contents to achieve cellular metabolic homeostasis (15). Intriguingly, autophagy is of vital importance in bone metabolism involving osteoblast-dominated bone formation, osteoclast-dominated bone resorption, and osteocyte-mediated bone matrix formation (13, 15). Defective autophagy in osteoblasts resulted in endoplasmic reticulum stress and induced significant bone loss, and GC treatment led to a decrease of LC3 (typical markers of autophagy)-positive osteoblasts (16, 17). In addition, activation of autophagy by Beclin-1 overexpression induced osteoclast differentiation and conditional ablation of ATG7 and treatment with chloroquine, an autophagy inhibitor, ameliorated GC-induced bone loss *via* inhibiting osteoclastogenesis (18). Besides, recent studies indicated that GC can induce bone fragility and reduce osteocyte numbers by suppressing autophagy (19). Therefore, the maladjustment of autophagy may have a relationship with the occurrence and progression of GIOP, but related studies are still insufficient.

In the present study, we provide evidence that ZGW aqueous extract may ameliorate GIOP *via* targeting let-7f and autophagy-associated genes (mTORC1, Beclin-1, ATG12, ATG5, and LC3). There was a possibility that both let-7f and autophagy might be involved in GIOP and were utilized as the therapeutic targets for ZGW aqueous extract in experimental GIOP rats.

## Materials and Methods

### Ethical Approval

The approvals of the animals and experimental procedures obtained access to the Ethics Committee of the First Affiliated Hospital of Guangzhou University of Chinese Medicine (GZUCM) (approval number: 20130425). Rats were fed in specific pathogen-free standard conditions which conform to the Guide for the Care and Use of Laboratory Animals, published by Institute of Laboratory Animal Resources (U.S.).

### ZGW Preparation

ZGW was purchased from the Beijing Tongrentang Pharmaceutical Factory (State Food and Drug Administration approval number: Z11020735). It comprised eight herbal medicines: Radix Rehmanniae (24 g), Semen Cuscutae (12 g), Radix Achyranthis Bidentatae (9 g), Carapax et Plastrum Testudinis (12 g), Cornu Cervi Pantotrichum (12g), Rhizoma Dioscoreae (12 g), Fructus Corni (12 g), and Fructus Lycii (12 g). Before the experiment, the drug was dissolved in normal saline, and a magnetic stirrer was stirred to help in dissolving. After dissolution, it was used for the animal experiment. For the cell experiment, ZGW solutions were filtered and sterilized at a 0.22-μm pore size for later use.

### Animals and Drug Administration

Three-month-old female Sprague-Dawley rats (n = 54) were obtained from the animal experiment center of Southern Medical University (Guangzhou, China), housed in the First Affiliated Hospital of GZUCM (SYXK [Yue] 2018-0092). The weight of rats at baseline was 220 ± 30 g. During the entire experiment, the rats were provided with food and water adequately.

After 1 week of adaptive feeding, the rats were randomly divided into three groups: the control group (CON) received vehicle intervention to eliminate systematic deviations caused by intervention factors; the dexamethasone (DEXA) group received subcutaneous injection of dexamethasone 0.6 mg/kg body weight twice a week for 3 consecutive months; and the ZGW group (ZGW) received oral administration of ZGW (1.62 g/kg), once a day for 3 consecutive months. The dose selection of ZGW was based on the recommended daily dose for humans (18 g/kg), and the equivalent conversion between animals and humans was based on the body surface area. This method of calculating the rat oral administration dose is similar to previous studies (9, 20).

### Sample Preparation

Rats in the CON, DEXA, and ZGW groups underwent euthanasia for the next experimental analysis at the end of the fourth week (M1, N = 6/group), eighth week (M2, N = 6/group), and twelfth week (M3, N = 6/group). Lumbar 2 (L2) specimens were separated for microcomputed tomography (micro-CT) and biomechanical analysis. L4 specimens were fixed with 4% paraformaldehyde for the next HE staining. Before assessing the AKP activity, blood samples were collected, centrifuged for serum, and stored at -80°C. Transmission electron microscopy was employed to detect autophagolysosomes from osteoblast L5 specimens. L6 specimens were stored at -80°C for real-time qPCR.

### Bone Microarchitecture

The software (μCT80 Evaluation Program v. 6.5-1; Scanco Medical, Brüttisellen, Switzerland) provided by the micro-CT system was employed to quantify the three-dimensional image and parameters of the L2 vertebral bodies, which was similar to previous studies (21, 22). The scanning conditions were 55 kV and 80 μA, and the spatial resolution was 14 μm. Vertebral cancellous bone was selected as the volume of interest. The microstructural parameters of cancellous bone were analyzed using micro-CT analysis software to determine the relative bone volume (BV/TV, %), structural model index (SMI), trabecular number (Tb. N), thickness (Tb. Th), and separation (Tb.Sp), and volumetric bone mineral density (vBMD). Three-dimensional images are obtained by reconstruction analysis.

### Bone Biomechanics Analysis

A material testing machine (ElectroPuls E1000 Testing System; Instron Corp., Norwood, MA) was employed for bone biomechanics analysis. Both end plates of vertebral bodies and their appendages were excised to form a central cylinder with parallel planes at both ends. The vertebrae were then examined along the longitudinal axis in this machine at a speed of 1 mm/min. Then the bone biomechanics parameters, including compressive stiffness, strength, and displacement as well as energy absorption capacity, were analyzed.

### Histological Analysis

L4 vertebral bodies were fixed with 4% paraformaldehyde for 48 h and then decalcified with 10% EDTA for 21–28 days. The samples were dehydrated with graded alcohol, embedded in paraffin, and then cut into 5-μm slices along the coronal plane using a paraffin microtome. Sections were stained with HE and observed under a microscope (BX53, Olympus Corporation, Tokyo, Japan).

### Alkaline Phosphatase Activity Assay

Rat serum AKP activity was analyzed using a kit (Nanjing Jiancheng Biological Products Co., Ltd., Nanjing, China). The serum alkaline phosphatase (AKP) levels in rats were determined using a visible spectrophotometer (Thermo Fisher, Waltham, MA, USA).

### Autophagolysosome Detection

The L5 samples were fixed with 0.2% glutaraldehyde at room temperature for 2 h, in 1% osmium tetroxide water for 1 h, and then stained in 2% uranyl acetate water for 1 h in the dark place. After dehydration through an alcohol gradient, the samples were embedded and sliced into 80-nm slices. The sections were stained with uranyl acetate and lead citrate and then observed with a transmission electron microscope (Philips CM, Amsterdam, Netherlands).

### Real-Time qPCR

The expression levels of let-7f, autophagy-associated genes (mTORC1, Beclin-1, ATG12, ATG5, and LC3), Runx2, and Cathepsin K (CTSK) were evaluated by RT-qPCR. Total RNA was extracted from L6 vertebrae or BMSCs using the TRIzol Reagent (Thermo, USA). The PrimeScript™ RT Master Mix Kit (Takara, Shiga, Japan) was used to reverse transcribe RNA to cDNA. Quantitative analysis was performed under a Bio-Rad qPCR instrument using the SYBR Premix Ex Taq™ II Kit (Takara, Japan). Actin or U6 was used as the internal standard for mRNA and miRNA, respectively. The relative expression of each gene was quantified using the 2^−ΔΔCt^ method. The primer sequences are shown in **Supplementary Table 1**.

### BMSC Culture, Osteogenic Differentiation, and ZGW Intervention

For osteoblast formation, we used rat bone marrow-derived mesenchymal stem cells (BMSCs). The separation and culture of rat BMSCs were performed as previously described (4). Briefly, we selected 7-day-old mice, dissected the femur in a sterile manner, and used a syringe needle to flush out bone marrow solutions. After centrifugation, the cell solutions were resuspended in α-MEM containing 10% fetal bovine serum (all from Gibco, Grand Island, NY, USA). The cells were cultured at 37°C in an incubator containing 5% CO_2_. Osteogenic differentiation was induced in rat BMSC osteogenic differentiation medium (Cyagen Biosciences, Santa Clara, CA, USA). After 7 days’ osteogenic induction, osteoblasts were fixed with 4% paraformaldehyde and stained using an BCIP/NBT Alkaline Phosphatase (ALP) kit (Beyotime, Shanghai, China).

### Luciferase Activity Assay

A PMIR-Report luciferase vector containing a let-7f binding site in the ULK2 3′-UTR was constructed. A mutant plasmid was used to measure binding specificity. Next, pGL3 luciferase reporter vectors were inserted into the let-7f promoter containing the ULK2 binding site. Then, we transfected luciferase vectors into osteoblasts. Luciferase activity was examined using the Luciferase Reporter Assay Kit (Promega, Madison, WI, USA).

### Western Blotting

BMSCs were lysed with RIPA lysis buffer (Beyotime) containing 1% protease inhibitor and 1% PMSF phenylmethanesulfonyl fluoride (PMSF) for 30 min to inhibit protein degradation and centrifuged at 12,000 rpm for 10 min, and the supernatant was transferred to a new tube. The BCA Protein Assay Kit (Beyotime) was used to determine the protein concentration; the Gel Preparation Kit (Beyotime) was used to perform SDS-PAGE. The separated proteins were transferred to polyvinylidene fluoride (PVDF) membranes (Millipore) and blocked with QuickBlock™ Western Primary Dilution antibodies (Beyotime) overnight at 4°C temperature. Anti-β-actin primary antibody (1:5,000, mouse, ab6276, Abcam, Cambridge, MA, USA), ULK2 (1:1,000; Rabbit; DF9890; Affinity, Cincinnati, OH, USA), ATG5 (1:1,000, rabbit, #12994, Cell Signaling Technology (CST), Danvers, MA, USA), ATG12 (1:1,000, rabbit, #4180, CST), Beclin1 (1:1000, rabbit, #3495, CST), and LC3 (1:1000, rabbit, #4108, CST) were used as the primary antibodies following incubation with the membranes for 12 h at 4°C. After washing the membranes with TBST sufficiently, the membranes were incubated with the corresponding secondary antibody for 2 h at room temperature and then washed three times with TBST. The protein level was determined by BeyoECL Moon enhanced chemiluminescence (Beyotime).

### Statistical Analysis

The data were analyzed with SPSS 19.0 software. For quantitative analysis, all results are expressed as mean ± standard deviations (SD). Statistical significance was evaluated by one-way analysis of variance (ANOVA) with the Student–Newman–Keuls (SNK) test for *post-hoc* analysis. Two-sided *p*-values < 0.05 were considered statistically significant.

## Results

### ZGW Improves Bone Quality of GIOP Rats

**Figure 1:**
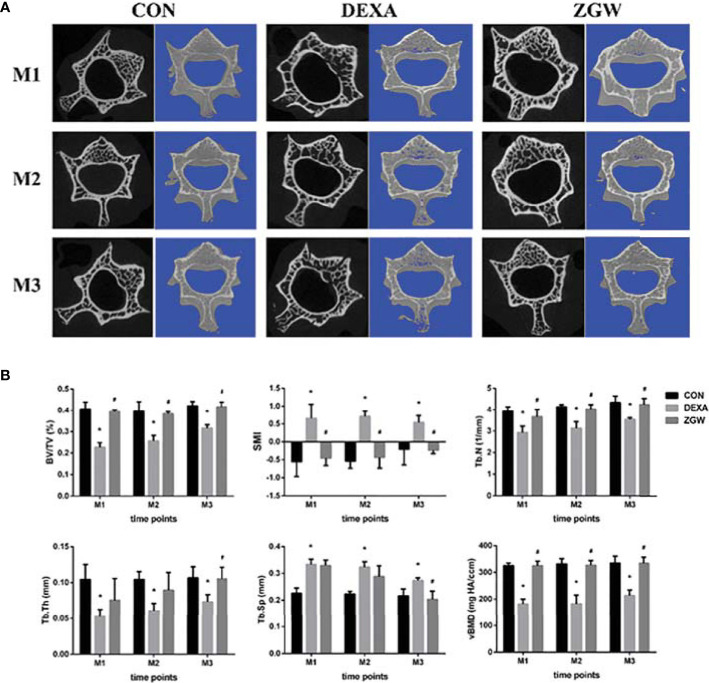
Effects of ZGW on bone microarchitecture and parameters determined by micro-CT. **(A)** Representative 2D and 3D micro-CT images. **(B)** Bone microarchitecture and parameters BV/TV, SMI, Tb.Th, Tb.N, Tb.Sp, and vBMD were calculated based on micro-CT results. Values are the means ± SD. ^*^p < 0.05 vs. CON group; ^#^*p* < 0.05 vs. DEXA group.

Micro-CT showed that there was a small amount of trabecular bone thinning and damage in the DEXA group at each time point, while the ZGW group had more trabecular bone and more dense distribution of trabecular bone **(** **Figure 1A****)**. Microarchitecture data of L2 cancellous bone of vertebrae examined by micro-CT displayed that the DEXA group showed low BV/TV, Tb.N, Tb.Th, and vBMD and significantly high SMI and Tb.Sp at all the time points compared to those in the CON group. Compared with the DEXA group, the ZGW group displayed increased BV/TV, Tb.N, Tb.Th, and vBMD at each time point and decreased SMI and Tb.Sp at each time point **(** **Figure 1B****)**.

### ZGW Increases Bone Strength of GIOP Rats

**Figure 2:**
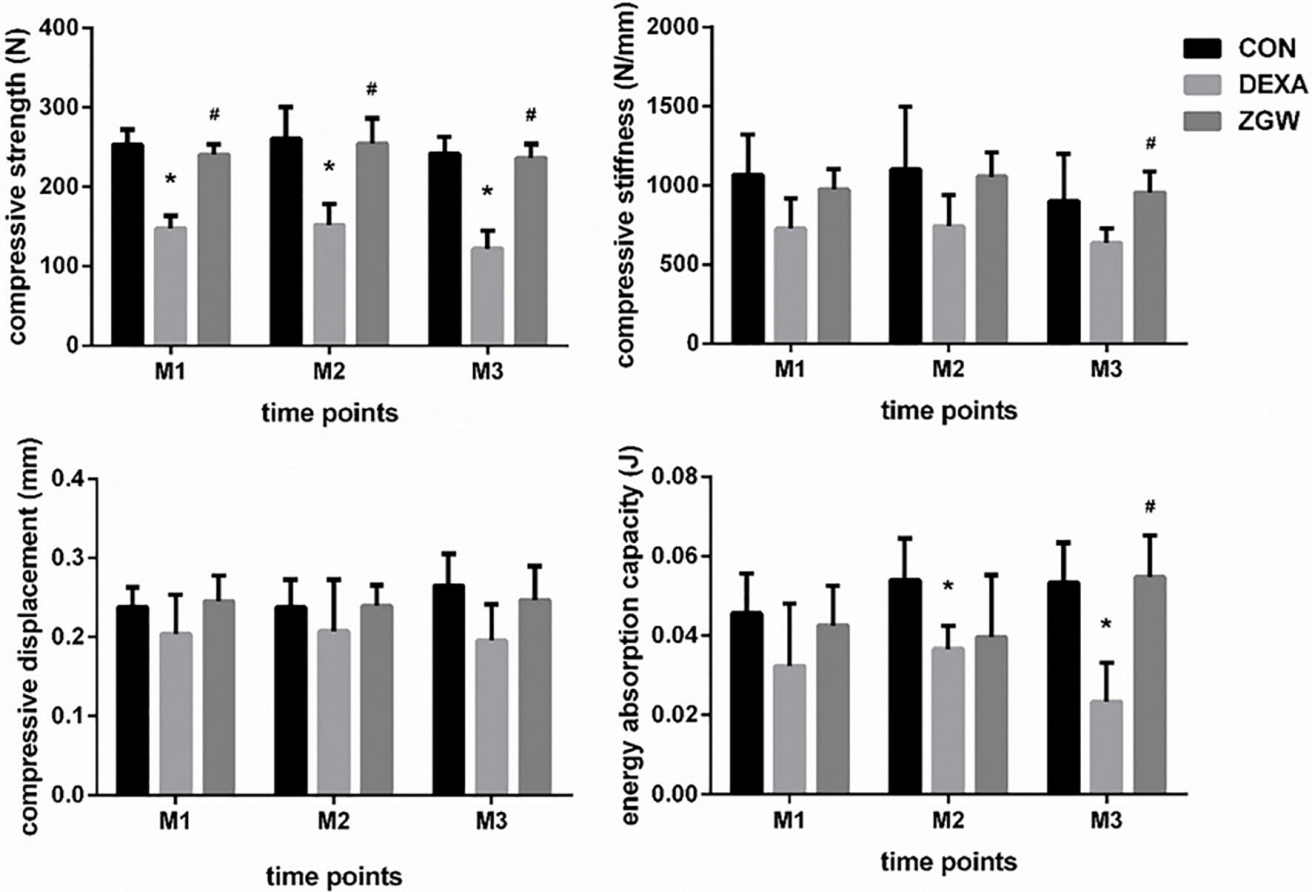
Results of biomechanical analysis of L2. Data are the means ± SD. ^*^p < 0.05 vs. CON group; ^#^*p* < 0.05 vs. DEXA group.

Compressive strength, compressive stiffness, compressive displacement, and energy absorption capacity were employed to evaluate the bone strength of GIOP rats. Compared to the CON group, the DEXA group displayed reductions in compressive strength at each time point and energy absorption capacity at M2 and M3. Meanwhile, the DEXA group (compared to the CON group) showed a decreased trend in compressive stiffness and compressive displacement at all the time points, although no significant differences were observed. Compared with the DEXA group, the ZGW group exhibited significantly increased compressive strength at each time point, high compressive stiffness, and energy absorption capacity at M3. Simultaneously, compared to the DEXA group, ZGW showed an increased trend in compressive stiffness at M1 and M2, compressive displacement at all the time points, and energy absorption capacity at M1 and M2, although the differences were not significant **(** **Figure 2****)**.

### ZGW Mitigates Histological Damage of GIOP Rats

**Figure 3:**
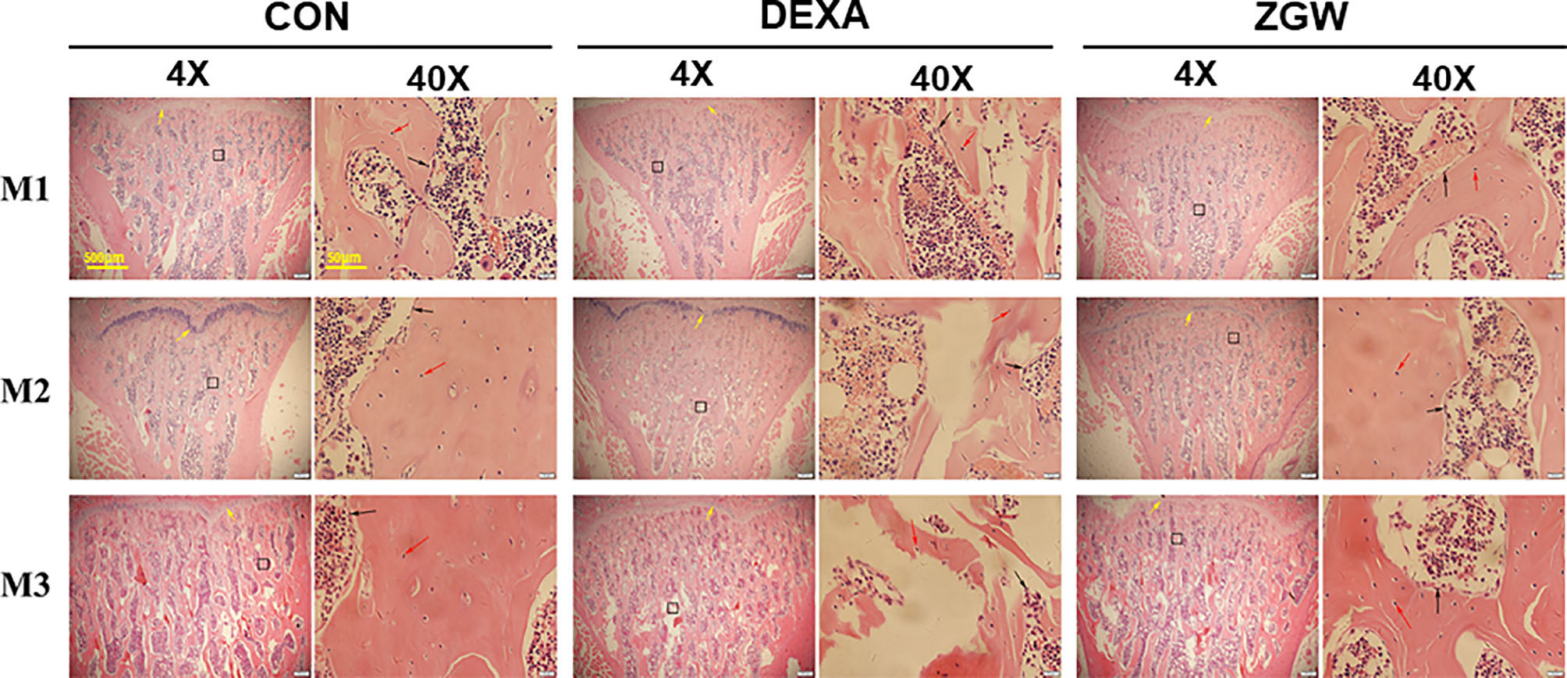
Changes of bone histomorphology examined by H&E staining in the L4 bone trabecula of rats in each group. Scale bars and magnifications are displayed on the images. The red arrows represent osteocytes; the black arrows represent osteoblasts.

HE staining was employed to assess the morphological changes in the L4 trabeculae of the various groups. At each time point, the DEXA group displayed increased trabecular spacing, trabecular absorption, perforation, fracture, and poor trabecular continuity compared to the CON group. Compared with the DEXA group, the ZGW group manifested improvements in vertebral trabecular bone **(** **Figure 3****)**.

### ZGW Increases Blood Serum AKP Activity of GIOP Rats

**Figure 4:**
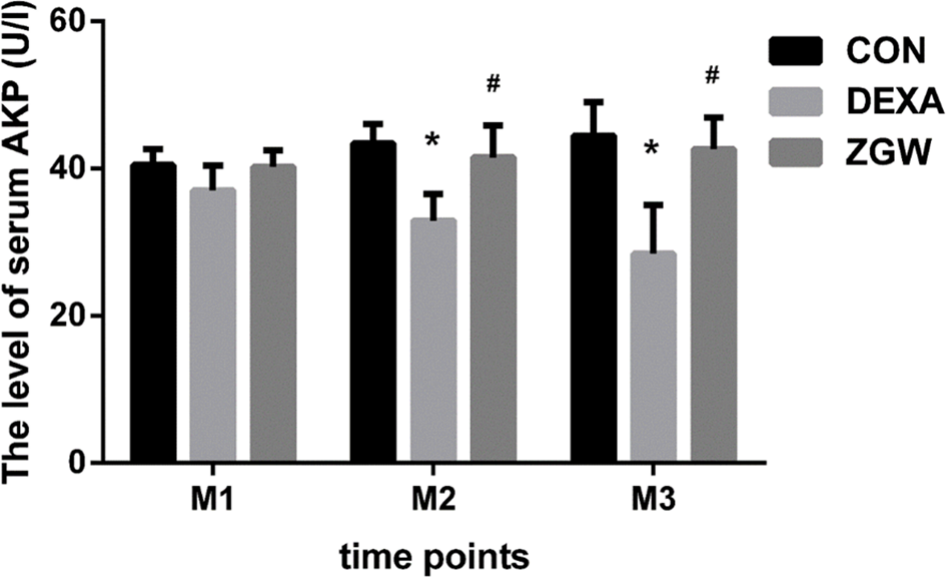
The levels of serum AKP. Data are the means ± SD. ^*^p < 0.05 vs. CON group; ^#^*p* < 0.05 vs. DEXA group.

ELISA was employed to detect the blood serum AKP activity of each group at different time points. Compared to the CON group, the DEXA group exhibited significantly decreased serum levels of AKP at M2 and M3. Compared with the DEXA group, the ZGW group manifested significantly increased serum levels of AKP at M2 and M3 **(** **Figure 4****)**.

### ZGW Decreases the Number of Osteoblast Autophagosomes

**Figure 5:**
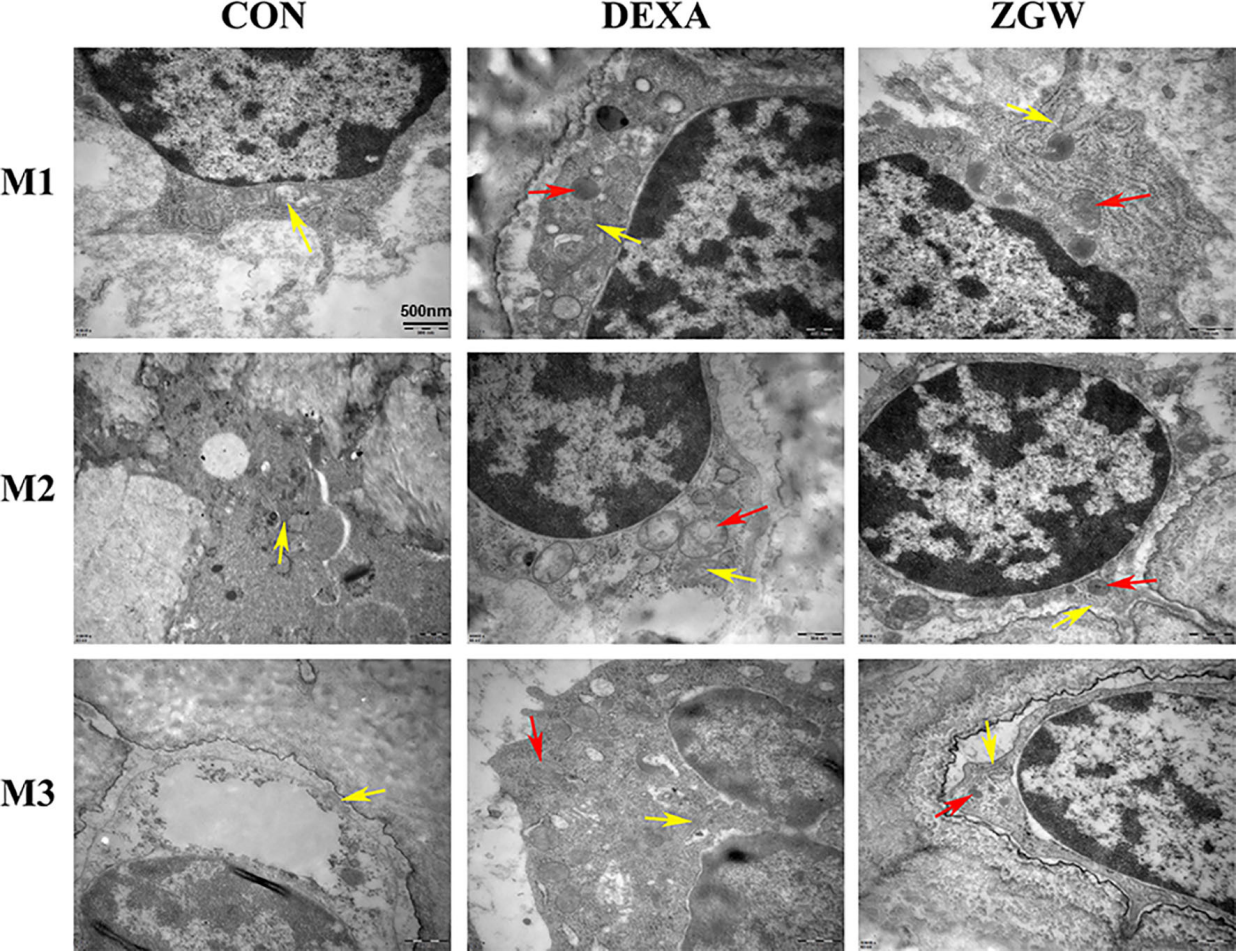
Transmission electron microscopic observation. The yellow arrows indicate mitochondria, and the red arrows indicate autophagosomes.

By transmission electron microscopic observation, at each time point, the DEXA group exhibited more cytosolic autophagosomes, which were abundant in the osteoblasts, than in the CON group. Compared with the DEXA group, the ZGW group showed a significantly opposite trend in autophagic characteristics **(** **Figure 5****)**.

### ZGW Promotes let-7f and Runx2 mRNA Expression, Increases Autophagy-Associated Negative Regulatory Gene mTORC1 Expression, and Reduces Autophagy-Associated Positive Genes (Beclin-1, ATG12, ATG5, and LC3) and CTSK mRNA Expressions

**Figure 6:**
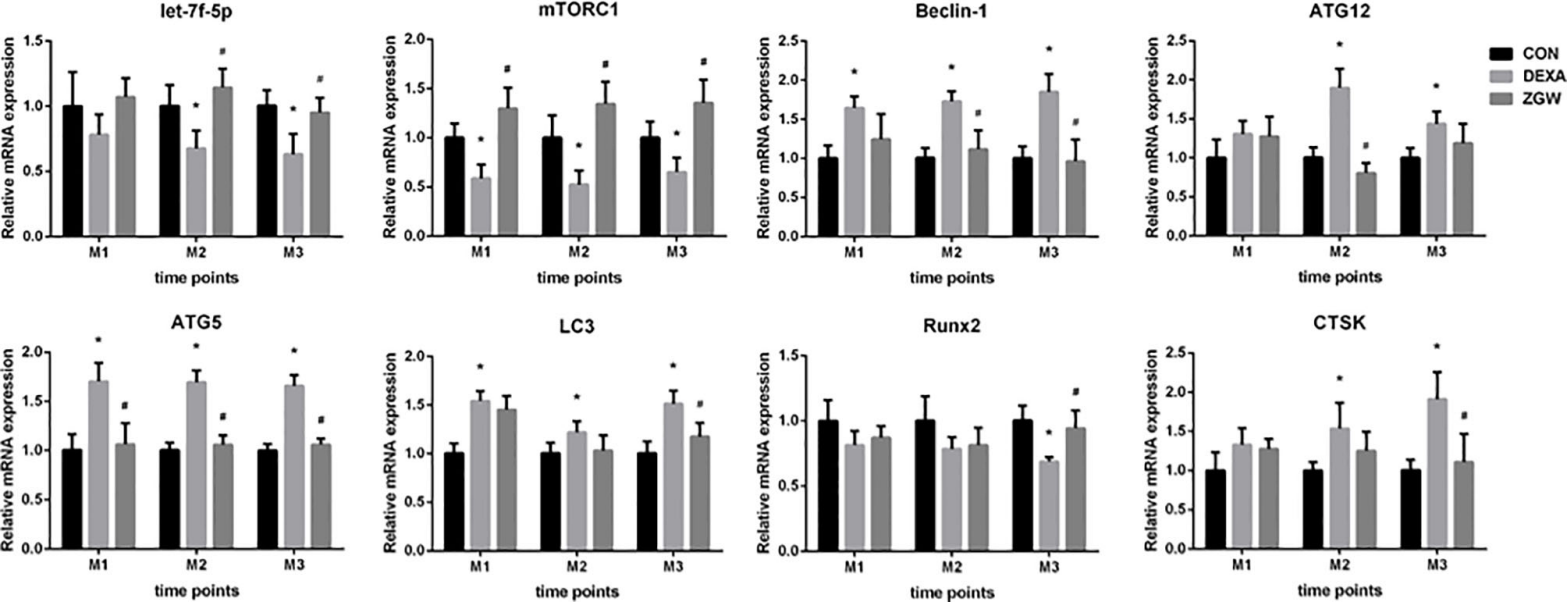
The mRNA expression levels of let-7f, autophagy-associated genes (mTORC1, Beclin-1, ATG12, ATG5, and LC3), Runx2, and CTSK. Data are the means ± SD. ^*^p < 0.05 vs. CON group; ^#^*p* < 0.05 vs. DEXA group.

The expressions of let-7f, autophagy-associated genes (mTORC1, Beclin-1, ATG12, ATG5, and LC3), Runx2, and CTSK were detected at the mRNA level to explore how ZGW might function. After DEXA intervention, let-7f expression was downregulated at M2 and M3. Furthermore, the DEXA group exhibited a significantly downregulated mTORC1 (a negative autophagy regulatory gene) mRNA expression at all the time points, significantly upregulated Beclin-1, ATG5, and LC3 mRNA expressions at all the time points, and ATG12 mRNA expression at M2 and M3. Meanwhile, bone formation-related gene Runx2 expression was downregulated at M3, whereas bone resorption-related gene CTSK mRNA expression was significantly upregulated at M2 and M3 **(** **Figure 6****)**.

After ZGW intervention, let-7f expression was significantly upregulated at M2 and M3 and was upregulated to some extent at M1 but did not differ significantly from that of the DEXA group. Compared to the DEXA group, the ZGW group exhibited a significantly downregulated ATG5 expression at all the time points, Beclin-1 expression at M2 and M3, ATG12 mRNA expression at M2, and LC3 mRNA expression at M3. Simultaneously, Runx2 expression was significantly upregulated at M3, whereas CTSK expression was significantly decreased at M3 **(** **Figure 6****)**.

### ZGW Partially Reverses the Decline of Osteogenic Differentiation Induced by let-7f Silencing

**Figure 7:**
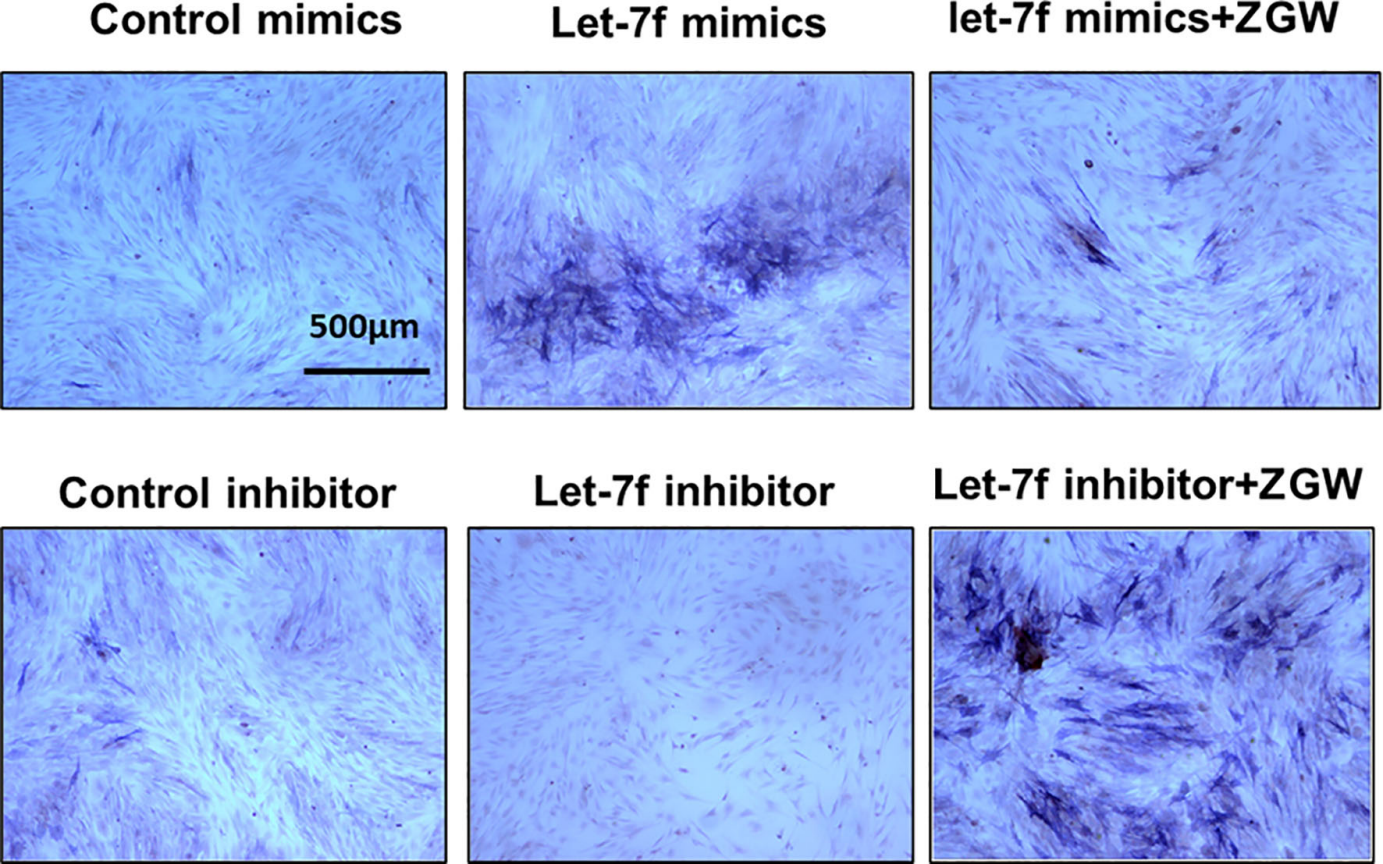
The osteoblast differentiation ability of BMSCs was evaluated by ALP staining. Scale bars are displayed on the images. Magnifications: ×4.

To explore the regulatory effect of ZGW on let-7f in osteogenic differentiation of BMSCs, we conducted gain-and-loss-of-function experiments by transfecting let-7f mimics of the inhibitor into BMSCs. The effect of overexpression or silencing was verified by qPCR **(** **Supplementary Figure 1A****)**. CCK8 was utilized to evaluate the effect of ZGW on BMSC proliferation. 10 μg/ml was the maximum concentration that did not affect BMSC proliferation, so we used 10 μg/ml ZGW as the optimum concentration in the next experiment **(** **Supplementary Figure 1B****)**. By ALP staining, we indicated that let-7f mimics could promote BMSC osteogenic differentiation, whereas the let-7f inhibitor suppressed BMSC osteogenic differentiation. After ZGW intervention, ZGW partially reversed the decline in osteogenic differentiation induced *via* let-7f silencing **(** **Figure 7****)**.

### let-7f Targets Autophagy Key Gene ULK2

**Figure 8:**
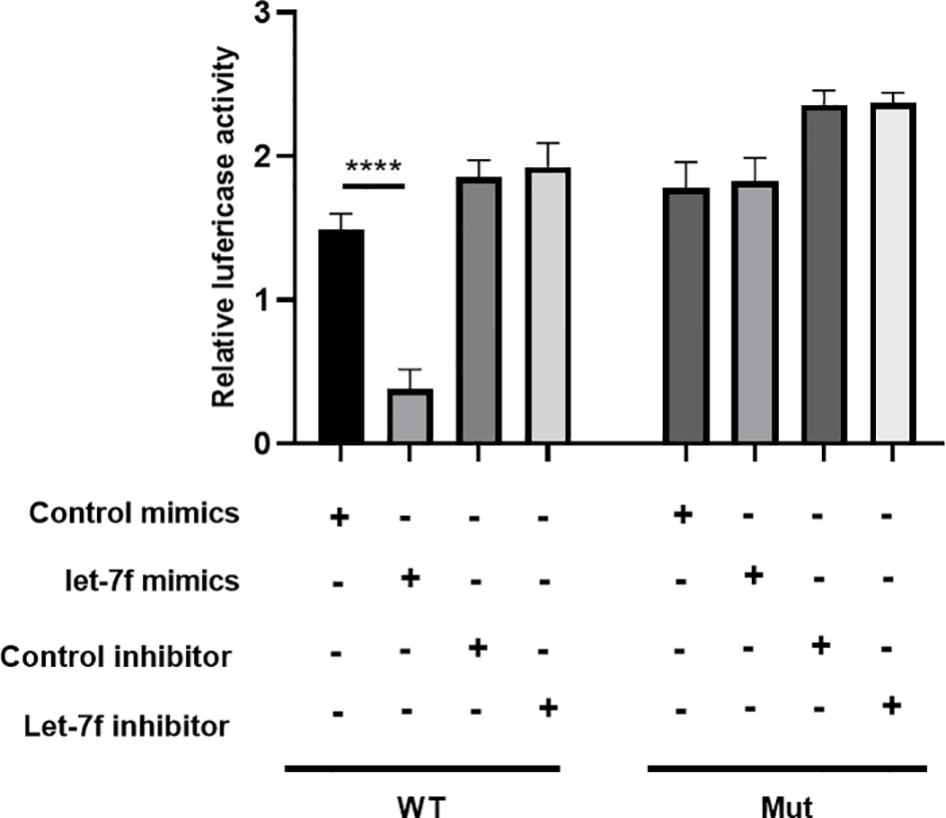
Luciferase reporter assay was evaluated the target relationship between let-7f and ULK2. ^****^p < 0.0001.

To examine whether let-7f binds to the 3′UTR of ULK2 (a positive autophagy regulatory gene), we conducted luciferase reporter assays by establishing a whole length of the ULK2 3′UTR, which was considered as the ULK2 wild-type (WT) 3′UTR. The ULK2 mutant (Mut) 3′UTR was established by binding sequence mutation. Following let-7f mimic transfection into BMSCs, the ULK2 relative luciferase activity was suppressed compared with that in WT (**Figure 8**). However, in the mutant (Mut) groups, the activity of ULK2 relative luciferase has no significant differences among the different groups. These data indicated let-7f in the regulation of ULK2 3′UTR **(** **Figure 8****)**.

### ZGW Suppresses let-7f Silencing-Induced Increased Autophagy

**Figure 9:**
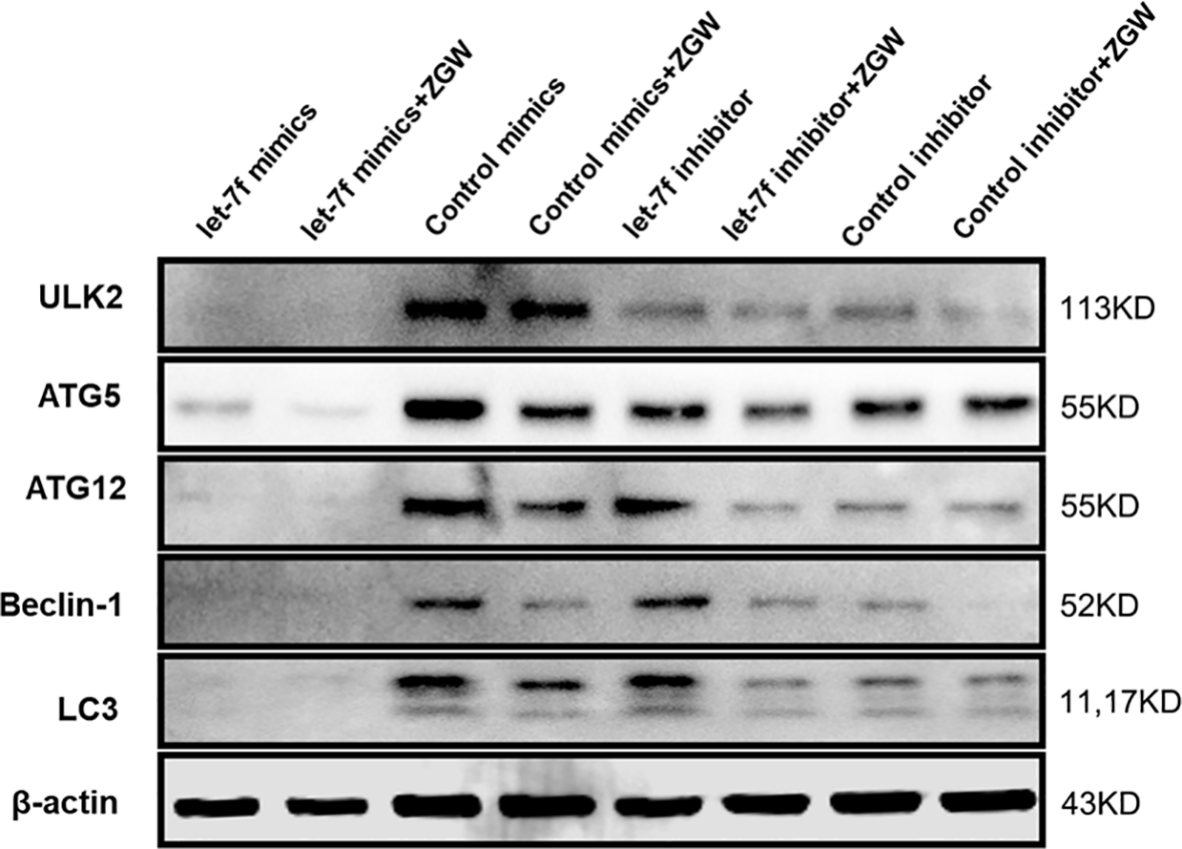
Changes in autophagy-related protein expressions (ULK2, ATG12, ATG5, Beclin-1, and LC3).

To further examine the effect of ZGW on let-7f in osteoblast differentiation of BMSCs, we employed Western blot to verify the relationship among let-7f, autophagy, and ZGW. We indicated that let-7f mimics could repress the autophagy-positive regulatory proteins (ULK2, ATG5, ATG12, Beclin1, and LC3) compared with the control mimic group. The let-7f inhibitor could increase the autophagy proteins, especially ATG12, Beclin1, and LC3, compared to the control inhibitor group; however, ZGW partially reversed let-7f silencing-induced increased autophagy **(** **Figure 9****)**.

## Discussion

The main pathological cause of GIOP is an imbalance of bone homeostasis between osteoblastic bone formation and osteoclastic bone resorption (23). Previous studies indicated that GCs resulted in bone impairment by reducing osteoblast differentiation, promoting the apoptosis of osteoblasts and osteocytes, and prolonging the life span of osteoclasts (2, 23). Furthermore, GCs can affect bone metabolism by regulating canonical Wnt-β-catenin and OPG/RANKL/RANK signaling, respectively (24). However, the precise pathogenesis of GIOP is not yet available and offering efficient therapy remains a challenge.

In the present study, we demonstrated that DEXA can result in severe bone damage, which was consistent with our previous studies (11, 25), indicating a success of the GIOP rat model. Simultaneously, our data showed that DEXA weakened serum AKP activity, which is recognized as an important marker of bone formation (26). We also verified that DEXA could increase CTSK expression, which is most abundant in osteoclasts (27), and decrease the expression of Runx2, an important osteoblast transcription factor, which was a benefit for bone formation (28), in GIOP rats. Moreover, DEXA induced the formation of autophagosomes in osteoblasts, suggesting that DEXA-induced excessive autophagy may be the key molecular mechanism of GIOP.

Intriguingly, ZGW, as an effective drug, had an ameliorative effect on GC-induced spinal osteoporosis of rats. Previously, some studies have indicated that ZGW was a promising prescription in the treatment of ovariectomy-induced bone loss (6, 9, 29). However, according to the authors’ current knowledge, the effect of ZGW on GIOP has not been reported. The effect of this Chinese herbal compound prescription was not only its ability to inhibit the expression of CTSK and impede bone resorption but also its ability to stimulate the expression of Runx2 and accelerate bone formation. Furthermore, the underlying mechanism of inhibiting bone resorption and accelerating bone formation with the ZGW aqueous extract was mediated, at least partially, through the activation of let-7f and regulation of autophagy-associated genes. Overall, ZGW may be a promising drug to prevent and treat GIOP.

**Figure 10:**
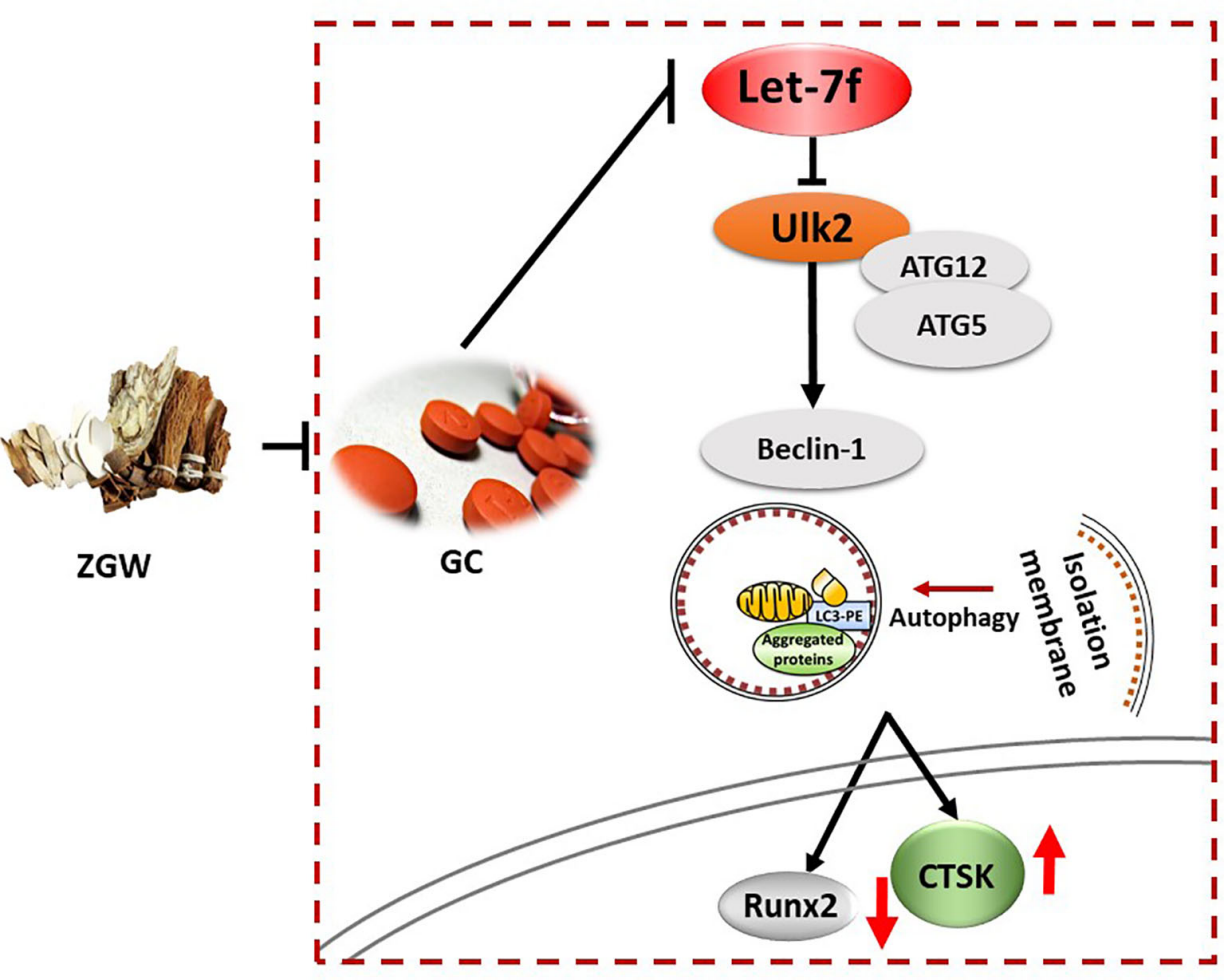
Mechanism graphic of the study.

Autophagy is characterized by the formation of autophagosomes by cell membrane encapsulation of part of the cytoplasm and organelles and fusion with lysosomes to form autophagolysosomes to degrade the encapsulated contents to achieve cellular metabolic homeostasis (13, 15). Autophagy promoters, such as GC, starvation, and rapamycin, can promote the formation of the ULK1/2 complex and increase the activity of the Beclin1–vps34 complex by promoting the phosphorylation of Ambra1 and Beclin1 (30, 31). Autophagosome elongation is mediated by two conjugated systems, LC3 and ATG12–ATG5–ATG16L, which are formed by the combined action of ATG7 and ATG10 (32, 33). In our work, *in vivo*, the expressions of Beclin-1, ATG12, ATG5, and LC3 were increased, while those of let-7f and mTORC1 were reduced in GIOP rats compared to those of the CON group. The aberrant expression levels of autophagy-related genes returned to the control value in the ZGW-supplemented GIOP rats. *In vitro*, we indicated that ZGW partially reversed the decline in osteogenic differentiation induced by let-7f silencing, the mechanism of which may be closely related to the activation of let-7f and suppression of autophagy. Therefore, we have reason to believe that ZGW could significantly ameliorate bone deteriorations in GIOP rats and promote BMSC osteogenic differentiation through activation of let-7f and suppression of autophagy **(** **Figure 10****)**.

## Conclusions

To our knowledge, for the first time, let-7f was selected to elaborate the pharmacological mechanisms for ZGW in experimental GIOP rats. Therefore, we have reason to believe that the ZGW aqueous extract could significantly ameliorate bone deteriorations in GIOP rats, and the underlying mechanism was mediated, at least partially, through activation of let-7f and suppression of autophagy. Our study provides an experimental basis for the clinical treatment of GIOP with traditional Chinese medicine. Moreover, it is beneficial to develop new drugs for treating GIOP concerning ZGW, let-7f, and autophagy-related targets. However, there are also some limitations in our study. First, the knockout and knock-in animals of let-7f are still necessary to further elucidate the mechanisms of ZGW in the treatment of GIOP. Besides, the efficacy of ZGW in the treatment of GIOP still needs to be confirmed by multicenter, double-blind, and randomized controlled clinical studies.

## Data Availability Statement

The original contributions presented in the study are included in the article/**Supplementary Material**. Further inquiries can be directed to the corresponding authors.

## Ethics Statement

The animal study was reviewed and approved by the First Affiliated Hospital of Guangzhou University of Chinese Medicine (approval number: 20130425).

## Author Contributions

Study design: GS, X,J and HR. Study conduct: GS, HR, QS, ZZ, HC, WZ, GC, XY, FY, PZ, JH, XZ, and LZ. Data collection and analysis: IM, JT, JC, and DL. Manuscript draft: GS, HR, QS. Manuscript revision: XJ. All authors contributed to the article and approved the submitted version.

## Funding

This research was funded by the National Natural Science Foundation of China (81904225 and 81774338), Innovation Team project of Guangdong Education Department (2021KCXTD017), Guangdong Natural Science Foundation (2020A1515110322 and 2021A1515011247), High-Level University Collaborative Innovation Team of GZUCM (2021xk57), and Natural Science Foundation of Xinjiang Autonomous Region (2019D01C013). The funding institutions had not any role in study design, data collection, data analysis, interpretation, or writing of the report in this study.

## Conflict of Interest

The authors declare that the research was conducted in the absence of any commercial or financial relationships that could be construed as a potential conflict of interest.

## Publisher’s Note

All claims expressed in this article are solely those of the authors and do not necessarily represent those of their affiliated organizations, or those of the publisher, the editors and the reviewers. Any product that may be evaluated in this article, or claim that may be made by its manufacturer, is not guaranteed or endorsed by the publisher.
