# Supplementary material for: Zuo-Gui-Wan Aqueous Extract Ameliorates Glucocorticoid-Induced Spinal Osteoporosis of Rats by Regulating let-7f and Autophagy
Source: Front Endocrinol (Lausanne). 2022 May 3;13:878963. doi: 10.3389/fendo.2022.878963 (PMC9111739; doi:10.3389/fendo.2022.878963)
Supplement: Supplementary file 1 [file DataSheet_1.docx]

**Supplementary Materials**

**Supplementary Figure 1**


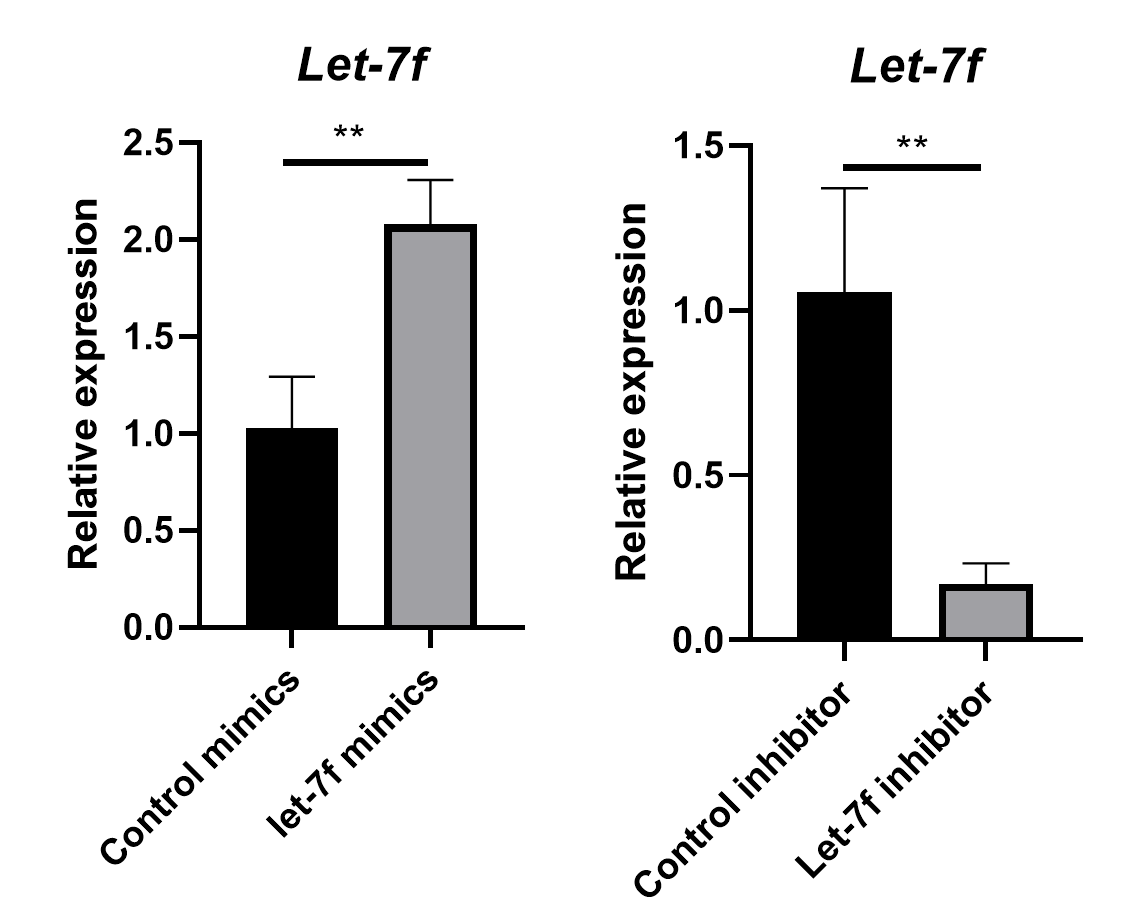


**Supplementary Figure 1.** The relaive expression levels of let-7f were identified by qPCR. Values are the means ± SD. ^**^*P* < 0.01.

**Supplementary Figure 2**


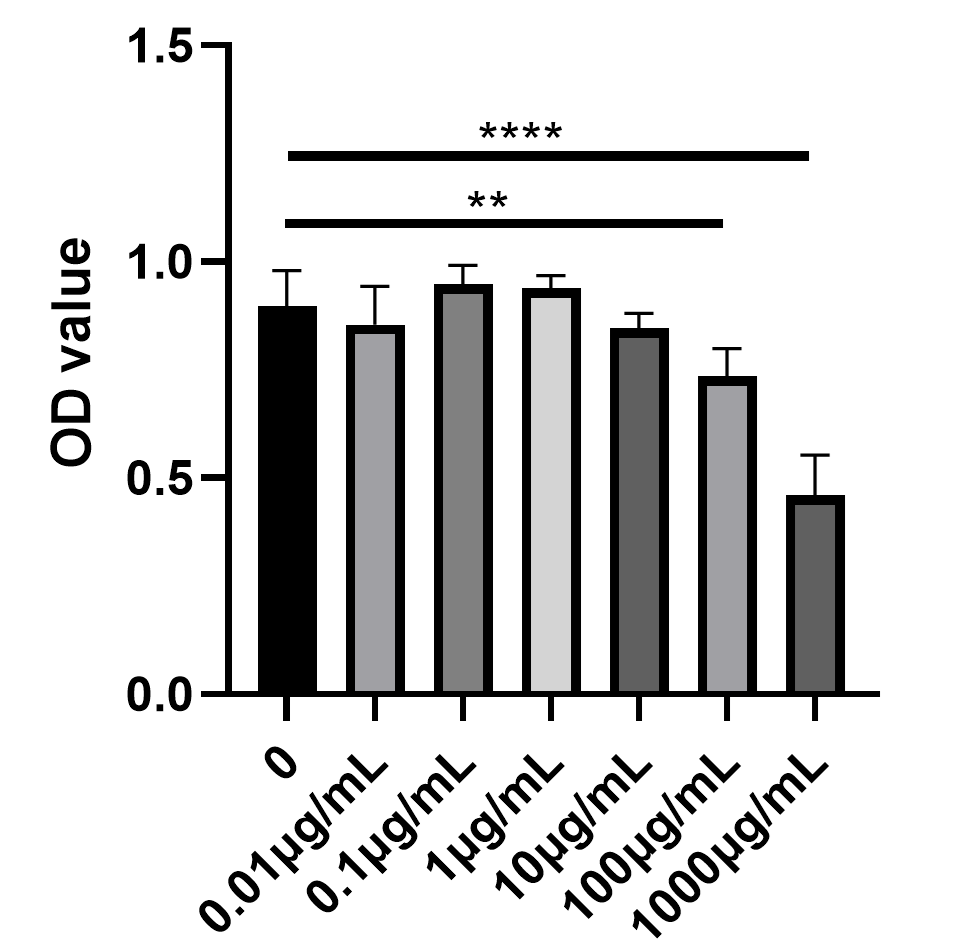


**Supplementary Figure 2.** Effect of ZGW on BMSCs proliferation was identified by CCK8. Values are the means ± SD. ^**^*P* < 0.01; ^***^*P* < 0.001.

**Supplementary Table 1. Sequences of primers**

| Gene | Primer sequence (5’-3’) |
| --- | --- |
| let-7f-5p-F | ACACTCCAGCTGGGTGAGGTAGTAGATTGTAT |
| let-7f-5p-R | TGAGGTAGTAGATTGTATAGTT |
| U6-F | TGCTTCGGCAGCACATATAC |
| U6-R | AGGGGCCATGCTAATCTTCT |
| mTORC1-F | GGTGGACGAGCTCTTTGTCA |
| mTORC1-R | AGGAGCCCTAACACTCGGAT |
| Beclin-1-F | CTCTCGT CAAGGCGTCACTTC |
| Beclin-1-R | CCTTAGACCCCTCCATT CCTCA |
| ATG12-F | CATTCTTACCTGGCGTTGAG |
| ATG12-R | CACTTCAAACCCTGTAATCC |
| ATG5-F | CCTGAAGACGGAGAGAAGAAGAG |
| ATG5-R | CGGGAAGCAAGGGTGTCAT |
| LC3-F | TGTTAGGCTTGCTCTTTTGG |
| LC3-R | GCAGAGGAAATGACCACAGAT |
| Runx2-F | CTTCCCAAAGCCAGAGCG |
| Runx2-R | CAGCGTCAACACCATCATTC |
| CTSK-F | CAGAGCGT-TACTCGCTTGGA |
| CTSK-R | GGTTGTGGAAACTCACACGC |
| Actin-F | GGAGATTACTGCCCTGGCTCCTA |
| Actin-R | GACTCATCGTACTCCTGCTTGCTG |
